# Linear programming based computational technique for leukemia classification using gene expression profile

**DOI:** 10.1371/journal.pone.0292172

**Published:** 2023-10-09

**Authors:** Mahwish Ilyas, Khalid Mahmood Aamir, Sana Manzoor, Mohamed Deriche

**Affiliations:** 1 Department of Computer Science & Information Technology, University of Sargodha, Sargodha, Punjab, Pakistan; 2 Artificial Intelligence Research Center, Ajman University, Ajman, UAE; HITEC University, PAKISTAN

## Abstract

Cancer is a serious public health concern worldwide and is the leading cause of death. Blood cancer is one of the most dangerous types of cancer. Leukemia is a type of cancer that affects the blood cell and bone marrow. Acute leukemia is a chronic condition that is fatal if left untreated. A timely, reliable, and accurate diagnosis of leukemia at an early stage is critical to treating and preserving patients’ lives. There are four types of leukemia, namely acute lymphocytic leukemia, acute myelogenous leukemia, chronic lymphocytic in extracting, and chronic myelogenous leukemia. Recognizing these cancerous development cells is often done via manual analysis of microscopic images. This requires an extraordinarily skilled pathologist. Leukemia symptoms might include lethargy, a lack of energy, a pale complexion, recurrent infections, and easy bleeding or bruising. One of the challenges in this area is identifying subtypes of leukemia for specialized treatment. This Study is carried out to increase the precision of diagnosis to assist in the development of personalized plans for treatment, and improve general leukemia-related healthcare practises. In this research, we used leukemia gene expression data from Curated Microarray Database (CuMiDa). Microarrays are ideal for studying cancer, however, categorizing the expression pattern of microarray information can be challenging. This proposed study uses feature selection methods and machine learning techniques to predict and classify subtypes of leukemia in gene expression data CuMiDa (GSE9476). This research work utilized linear programming (LP) as a machine-learning technique for classification. Linear programming model classifies and predicts the subtypes of leukemia Bone_Marrow_CD34, Bone Marrow, AML, PB, and PBSC CD34. Before using the LP model, we selected 25 features from the given dataset of 22283 features. These 25 significant features were the most distinguishing for classification. The classification accuracy of this work is 98.44%.

## Introduction

Blood is the most important component of the human body, consisting of 55% liquid termed plasma that flows freely through a blood vessel. Plasma primarily aims to transport nutrients, proteins, and hormones to and remove waste from the human body. Red blood cells (RBCs), white blood cells (leukocytes), and platelets are the three biological components that can be distinguished by their colour, shape, size, texture, and content (thrombocytes) [1]. When certain blood cells die, the blood transports less oxygen, which causes fatigue and weakness. Because blood cells are abundant, an excess or deficiency of any blood cell causes a variety of health issues, including leukemia, sickle cell disease, thalassemia, and anaemia. Leukemia develops due to an excess of WBCs in the immune system, which crowds out the healthy RBCs and platelets of blood. Leukemia is one of the most frequent diseases that can result in mortality. To overcome the severity of this disease, diagnosing the forms of immature cells at an early stage is vital, which minimises the patients’ modality rate. Many researchers have proposed approaches and algorithms for detecting, segmenting, and classifying leukemia. A precise early-stage leukemia diagnosis is essential for treating patients and their survival. The four types of leukemia [2] are acute lymphocytic leukemia (ALL), acute myelogenous leukemia (AML), chronic lymphocytic leukemia (CLL), and chronic myelogenous leukemia (CML). Recognising these cancerous development cells is often done manually via microscopic image analysis and requires an extraordinarily skilled pathologist. A professional pathologist collects a blood sample to detect these cells, which are then stained on a blood slide. Staining allows cells to be examined under a high-quality microscope to detect morphological characteristics of many components of blood cells, such as WBC, RBC, platelets, parasites, blasts, and any other anomalous condition. This identification and diagnosis are crucial and require a qualified and experienced pathologist to conclude. As a result, providing an automated method for this diagnostic is always necessary and required. Microarray technology offers a way to concurrently track hundreds of genes’ levels of RNA expression in primary tumours and cell lines. Making a diagnosis is difficult for medical practitioners since they must consider a wide range of clinical data aspects. Because of the difficulties in diagnosing leukemia, all medical professionals seek to remove uncertainty by acquiring precise information to treat the patients’ various conditions. Diagnosis, a clinical decision-making process, provides valuable information for improving healthcare quality [3].

Researchers may now simultaneously study thousands of genes that make up a significant part of the genome using microarray technology. The development of this promising technology has generated interest in its prospective uses in clinical diagnostics and pharmacological research. Finding gene subsets, sometimes referred to as biomarkers that distinguish between occurrences with different labels, such as different tumor types, cancer vs. non-cancer, and therapeutic response, is a vital step in these kinds of applications. A supervised learning task is the first that tries to accurately determine its label, such as whether it is either a tumor or normal tissue, given a gene expression pattern. Over the past few years, this strategy has been successfully tested with several algorithms [4] and has several applications in clinical diagnostics. Gene selection, the other objective for linear programming is a subclass of the broader issue of feature selection (FS) that is a dimensionality reduction technique [5]. The dimensions are chosen by FS, yet, by merging the many input dimensions, feature extraction techniques like Principal Component Analysis (PCA) minimize dimensionality. This is important for gene selection because it preserves the physical meaning of the genes, facilitating interpretation [6], and the features are the genes’ expression values. On gene declaration information, highlight extraction and component selection procedures are time-consuming for dimensionality reduction, information perception, as a phase in the readiness of different calculations, or to recognize a subset of more significant genes. The study of gene expression data is motivated by the challenge of classification between cancer classes or finding multiple subclasses of malignancies. However, a variety of factors could have an impact on the analysis’s findings. The curse of dimensionality which is brought on by small sample sizes in comparison to high numbers of characteristics, is one of the biggest problems. The generalizable performance of a classifier suffers from having too many characteristics, some of which may be unimportant to analysis. Choosing selective genesis is therefore crucial to enhancing the precision and speed of prediction systems [7]. Choosing an appropriate feature set is critical for developing effective and efficient models, improving comprehensibility, minimising overfitting, and reducing complexity.

This research is based on linear programming classification of leukemia subtypes that accompany cancer diagnosis. Linear programming (LP) approaches may be able to quickly and precisely identify expression patterns. Microarray data has typically been subjected to linear programming with the two different but complementary goals of sample categorization and gene choice. This study uses feature selection and linear programming techniques to classify types of leukemia based on leukemia gene expression data. Given that the dataset used to evaluate gene expression levels had 22283 genes (columns) from 64 samples, and that this was too large, utilizing a feature selection strategy improved the prediction technique’s effectiveness (rows). As a result, reducing the data quantity contributed to improving classification performance.

Our classification study is useful to classify the different subtypes of leukemia represented in chosen dataset GSE9476 on leukemia gene expression from CuMiDa through feature selection methods and machine learning techniques.

The remainder of the paper is organised as follows: The literature review is discussed in section II, the proposed methodology with its steps is discussed in section III, the results are discussed in section IV, and the conclusion and future work is discussed in section V.

## Literature review

Many researchers have identified and predicted different cancer subtypes using different types of methods. This section discussed the most notable research that made use of gene datasets via machine learning including Linear programming-based leukemia subtypes research. Y. Tang et al. [4] developed an FCM-SVM-RFE Recursive Feature Elimination (RFE) algorithm for predicting AML/ALL gene expression data, which achieved an average accuracy of 92.94%. The Fuzzy C-Means clustering approach was used to group related genes into clusters, and then a Support Vector Machine (SVM) was modeled in each cluster-induced space. This method was more accurate for predicting unknown samples of cancer [2]. Yoo et al. [8] suggested a gene selection and multivariate fuzzy statistical analysis technique for evaluating microarray data from leukemia patients. It was used to analyze the gene expression pattern and investigate the leukemia subtypes whose expression patterns were found to be linked to the cases of acute leukemia gene expression. They used PCA to evaluate ALL and AML patterns. It also eliminates the drawbacks of threshold-based gene selection, such as the impossibility of an unknown subclass selection. Taskesen et al. [9] worked on bringing gene expression profiles (GEP) and DNA methylation profiles (DMP) together. Gene expression profiles, as well as the gene patterns obtained from GEP, can be utilized to predict AML subtypes. Similarly, DNA-methylation profiles were used to make successful predictions. Both have different patterns that aid in the classification of AML subtypes. They employed a logistic regression model with Lasso regularization to predict AML subtypes. He et al. [10] worked on classification methods for leukemia cancer. To efficiently extract high-level data abstraction and transform this quantitative data into fuzzy discrete transactions, authors combined data clustering approaches with fuzzy interval partitioning on given features. These transactions were supplied to the A priori algorithm to mine association rules that supported better classification and decision. Experiments reveal that the FARM-DS mining technique for Fuzzy Association Rules (FARs) has good interpretability since it extracts considerably shorter rules and has great prediction accuracy. Klein et al. [11] presented a novel approach for systematic and rigorous comparison of published gene expression identifiers to a demonstrative given dataset. Identifying related analyses and gene mutations, enhanced the analysis of new microarray data. This technique enables researchers to integrate learnings from multiple microarray experiments into the structured analysis of a new dataset. Stiglic et al. [12] introduced a new method for interpreting tiny ensembles of classifiers using gene expression data called Visual Interpretation of Small Ensembles (VISE). It was proven that interactive interpretation tools, which were created for traditional machine learning challenges, also provide a wide variety of opportunities for researchers in the bioinformatics discipline. They also serve as an interactive tool for experts in the classification process. Feltes et al. presented Curated Microarray Database (CuMiDa) which is a resource that contains 78 cancer Microarray datasets that have been rigorously cross-checked from 30,000 Gene Expression Omnibus (GEO) articles. CuMiDa is a database of datasets dedicated to the testing and benchmarking of machine learning algorithms in cancer research. Feltes et al. observed sample division for this, all data sets were tested using principal component analysis (PCA) and t-distributed stochastic neighbor embedding (tSNE) analyses, as well as various machine learning (ML) approaches including SVM and RF, to provide a base accuracy of 88–85% for the major techniques used for microarray data sets [13]. Bilen et al. have developed a new method for rapidly classifying leukemia cancer microarrays and decreasing data size by focusing on the most important genes. Bilen et al. employed two methods, the ensemble, and the hybrid method. Firstly, a gene filtering algorithm is created using the Wilcoxon rank-sum, Fisher correlation score, and information gain approach to create an ensemble gene selection algorithm. Secondly using an upgraded genetic algorithm, the most successful genes among these genes are exposed in the feature selection phase. Cross-validation findings after the classification process were 100% (LOOCV), 98.57% (5-fold), and 97.14% (10-fold) [14]. To categorize microarray data with a small sample size and a large number of features, Xu et al. used two Modified Linear discriminant analysis techniques. Xu et al. mentioned the reason behind the sub-optimal performance of classical LDA on microarray data in terms of uncertainty and uniqueness of the within-group covariance matrix. The MLDA and NLDA have been used in their study and analyzed that modified LDA techniques work better in classifying data that has a large number of features and small samples when compared with the k-nearest neighbor, diagonal linear discriminant analysis, and classical LDA [15]. When working with high-dimensional data that has a little quantity of labeled data and a significant number of unlabeled data, it is never easy to get better classification results. A semi-supervised sparse Fisher’s LDA was proposed by Lu and Qiao. LDA is rebuilt and sparsity is attained using a direct estimation technique. To deal with the no convex loss function related to the unlabeled data, they additionally employ the difference-convex approach. Overall, the suggested strategy improves the LDA method’s capability [16]. Feltes et al., manually curated the Gene Expression Omnibus GEO using extensive filtering parameters to select the major homogeneous and high-quality RNA-seq using microarray datasets having several cancer types. TCGA data was used to study frequently unregulated genetic mechanisms behind the tumoral process using machine learning techniques and biological processes. His findings showed that tumor is more closely linked to the overexpression of essential unregulated machinery than to the under-expression of a specific gene [13]. Zhou et al. has been used Neural networks, Bayesian statistics, and a self-organizing map in research. In Microarray datasets, neural networks are best for feature learning and computation. When compared to large feature scales, the sample sizes are found to be insufficient. Because dealing with such high-dimensional, small-sample-size data is tough, a combination of BNN and SOM can perform well, particularly in classification problems involving gene expression-related disorders. The self-organizing map is best for dimension reduction, whereas Bayesian statistics are used to estimate feature ambiguity from the posterior distribution [17]. Grisci et al. worked on a novel strategy that uses Neuroevolution as a machine-learning method to classify microarray data and choose more relevant genes at the same time. The author used the FS-NEAT algorithm. In addition, quality microarray datasets were selected using a strict filtering and preprocessing approach. When evaluated with microarray datasets of three different forms of cancer with variable numbers of samples, characteristics, and classes, the Grisci et al. approach reduced the number of dimensions in all datasets by over 99.9%. The use of the features chosen by his method improved the performance of algorithms [18]. Liu et al. used basic particle swarm optimization (PSO) to identify acute leukemia samples with 96.43 percent accuracy. It was compared to K-means clustering, and the findings showed that PSO performs better than K-means, but stability is flipped [19]. Karim et al. introduced a deep learning-based gene expression data classification method that used the Grey Wolf Optimizer (GWO) to train Sparse Auto-Encoders via an unsupervised training process. Auto-Encoders (AE) has a unique property that allows them to extract high-level attributes from row data, and thus they achieved 98.99% accuracy. Under the same test conditions and for the same datasets, in this research, the GWO method results has been compared with some other against extensive Meta heuristic algorithms such as Particle Swarm Optimization (PSO), Artificial Bee Colony (ABC), and Genetic Algorithms (GA). Sparse Auto Encoders trained on GWO outperform both conventional approaches and the abovementioned techniques [20]. Sun et al. [21] proposed a model by using gene expression patterns for cancer and other gene-disease categorization. This field of clinical diagnosis is becoming increasingly important for accurate cancer diagnosis and the identification of cancer subtypes. To increase the accuracy of microarray data outputs, authors suggested a gene selection strategy based on Fishers Linear Discriminant (FLD) and neighborhood rough set (NRS). Fisher’s Linear Discriminant technique was useful in reducing generic data so that a potential gene subset with strong classification capability could be obtained. After that, Sun et al. worked on defining neighborhood roughness and precision in a neighborhood decision system. Experiments showed that Sun et al. proposed a strategy that can pick a smaller and well-classified gene sample and improve classification results. W. Tang et al [22] proposed a novel compressive sensing (CS)-based technique for leukemia subtyping to classify ALL and AML. The CS method, a new technique for computational and statistical signal analysis, allows signals to be recovered from a small number of incoherent projections. To determine the class, the LOO method was used, which allows for signal reconstruction from a small number of incoherent signal projections. It uses fewer computations and resources and achieves 97% classification accuracy.

Silva et al. compared three distinct machine-learning models and data mining techniques to diagnose acute myeloid leukemia and acute lymphoblastic leukemia on gene expression data. The primary algorithm was the support vector machine, the second was the artificial neural network, and the third was the machine learning ensemble, which is a collection of various intelligence algorithms (Artificial Neural Network, Support Vector Machine, Random Forest, Gradient Boosting, and k-NN). The learning ability and classifying potential of the Ensemble model were consistent, and it performed better than 94% in classifying AML and ALL leukemia types [23]. CuMiDa is a valuable resource in cancer biomedical research for benchmarking machine learning techniques in leukemia gene expression analysis [24, 25]. The existing literature has been summarized in Table 1.

**Table 1:** A summary of the significant leukemia classification research work based on gene datasets.

Ref.	Author & Year	Methodology	Study Area	Results
[8]	Yoo et al., (2005)	Multivariate Fuzzy Statistical Analysis	Interpreting patterns and analysis of acute leukemia	Evaluated ALL and AML patterns. Using 25 genes selection
[9]	Taskesen, et al.,(2015)	LR Model with LassoRegularization	Molecular subtype classification in AML	Improved prediction power
[10]	He et al., (2006)	FARM-DS	Mining fuzzy association rules for leukemia classification.	Better classification and decision support.
[11]	Klein et al., (2009)	Similar Studies and Gene Mutations	Quantitative comparison of microarray experiments	Combined multiple microarray experiments.
[12]	Stiglic et al., (2007)	Visual Interpretation of Small Ensembles	Gene Expression Analysis of Leukemia Samples	Developed an interactive tool in the classification process.
[14]	Bilen et al., (2020)	Enhanced Genetic Algorithm	Classification of Leukemia Cancer	Rapid classification of leukemia cancer, Accuracy 97%
[15]	Xu et al. (2009)	Modified Linear Discriminant Analysis	Classification of high dimensional data	Analyzed that modified LDA techniques work better in classifying
[16]	Lu & Qiao, (2018)	Semi-Supervised Sparse Fisher’s LDA	Rebuilt LDA	To deal with the nonconvex loss function employed the difference convex approach.
[13]	Feltes et al., (2019)	PCA & t-SNE(SVM-RF)	Benchmarking and Testing of ML Approaches in Cancer Research	Provided base accuracy for major ML techniques. 88–85%
[17]	Zhou, (2021)	Bayesian statistics + NN (BSOMs and DSOMs)	Gene-Based Disease Classification	Better feature learning. BSOM = 76.13%, DSOM = 78.99%
[18]	Grisci et al., (2019)	FS-NEAT	Pattern Identification in Cancer Research	Improved the performance of algorithm, Results achieved 93%
[19]	Liu et al., (2012)	Particle Swarm Optimization	Classification of Leukemia Gene Expression Data	Identification of Acute leukemia. 96.43%
[20]	Karim, (2022)	Grey Wolf Optimizer (GWO) Algorithm	Data Classification	Trained Sparse Auto-Encoders via an unsupervised training process. Results achieved 98.99%
[21]	Sun et al., (2018)	FLD) and neighborhood rough set	Gene selection approach	Demonstrated a neighborhood decision system reduction model and validated it.
[22]	Y. Tang et al., (2005)	FCM, SVM, RFE	Leukemia Classification from Microarray	Accurate prediction of unknown
[23]	Silva et al. (2021)	ML Ensemble	Diagnose AML and ALL leukemia types.	Better classification, Results 94%
[26]	Xie et al. (2017)	Hierarchical Clustering Analysis	Bipartite network analysis of AML	Improvement in cancer prognosis. The hazard ratio for training (HR) = 1.58, *p* = 0.038) for testing (HR = 1.69 and 1.56 and *p* = 0.089 and 0.029)

## Proposed methodology

**Fig 1:**
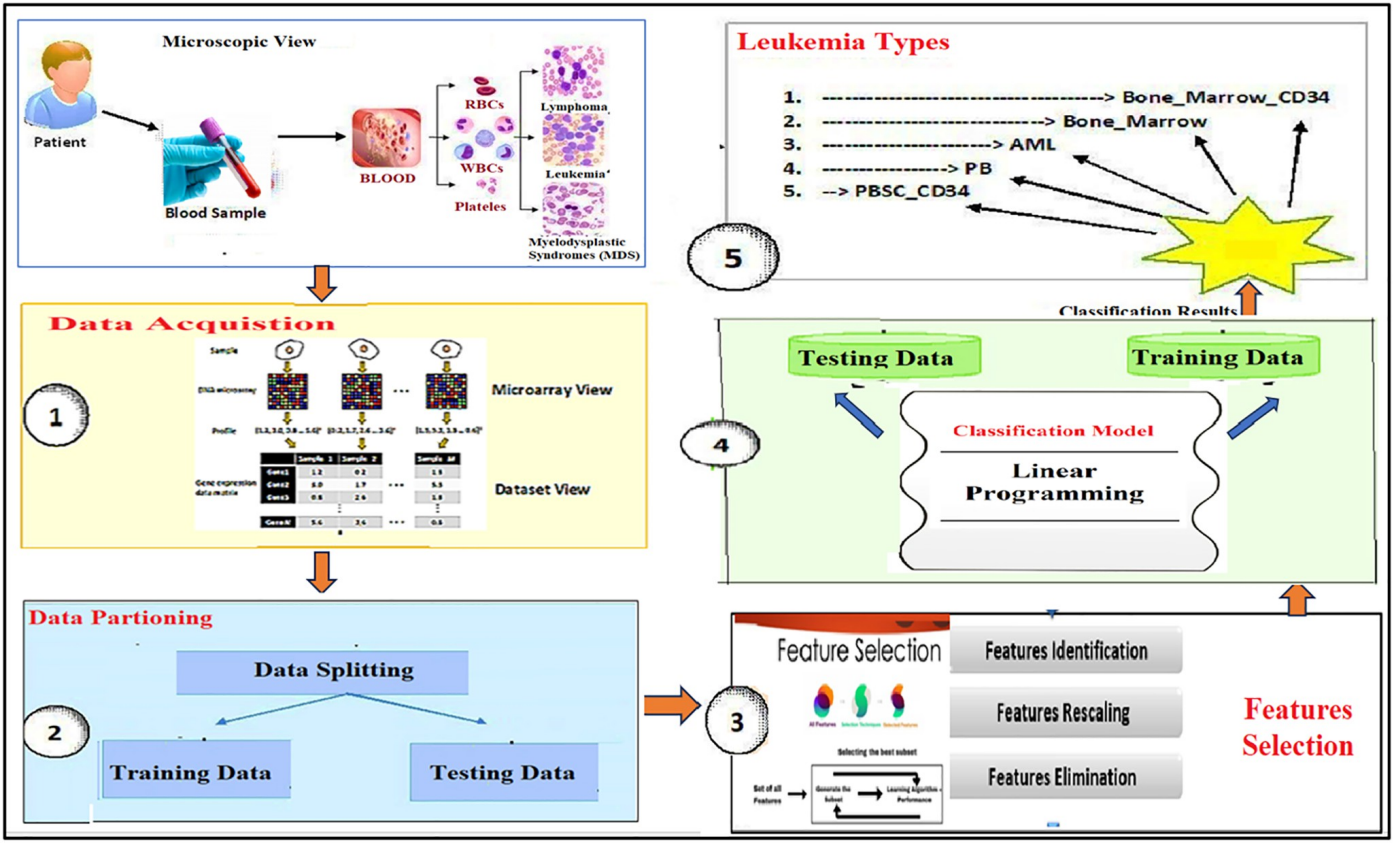
Proposed methodology for the leukemia classification.

The proposed methodology for the classification of leukemia consists of the following steps including data acquisition, data preparation, feature selection, and design of the classifier. A detailed flow diagram of the proposed model is given below in Fig 1.

### Data acquisition

The datasets Leukemia gene expression–CuMiDa [27] has been used in this methodology for the classification of leukemia. The details of the selected datasets are given in Table 2.

**Table 2:** Description of the selected dataset CuMiDa.

Dataset	Dataset GSE9476
Name	Leukemia gene expression–CuMiDa
Author	Bruno César Feltes
Classes	5
Samples	64
Features	22283
Type	Numeric
Source	Kaggle
Repository	CuMiDa^10^

In this dataset, there is a total of 64 leukemia samples including 8 cases of Bone_Marrow_CD34, 10 cases of Bone_Marrow, 26 cases of AML, 10 cases of PB, and 10 cases of PBSC_CD34. It consists of 5 classes, these five types include Bone_Marrow_CD34, Bone_Marrow, AML, PB, and PBSC_CD34.

### Data preparation

After acquiring the dataset the next step was understanding its features and types. The chosen Dataset GSE94769 is a numeric dataset. We must first display the dataset to understand the behavior of the characteristics and make predictions regarding anomalies. For this, we used *MATLAB R2021a* environment for performing tasks and operations on our dataset. This study was implemented by using the open-source platform MATLAB. We displayed the whole data for each class to identify any outliers or abnormalities. Additionally, visualization greatly aids in data understanding and interpretation, allowing us to use the proper machine-learning techniques and algorithms to create computationally robust models. The data is uniform and has few outliers. Leukemia data is split into two parts, testing and training data.

### Feature selection

As the microarrays hold enormous potential for accelerating the discovery of new biological information since they can concurrently measure the expression levels of thousands of genes. One characteristic of microarray data is that there are many more variables ’P’ (genes) than sample size ’N’. As in the case of our dataset, there are N = 64 samples and P = 22283 genes in total. In limiting the size of the feature set, thus we must find an appropriate gene selection strategy for our microarray dataset so that the feature size should be reduced. Total 25 data features have been obtained from leukemia gene expression dataset. These are the most distinguishing features that are useful for classification. As biomedical problems related to genes are complex and it is difficult to build a perfect model, the ideal case gives near about 100% classification accuracy. Different feature extraction techniques were recommended in the literature to improve classification rates and lower processing costs for identifying important genes. So, we improved our results by extracting those features that give better data separability. Reducing the features helped improve prediction performance in terms of speed and accuracy. The process of choosing the best suitable subset of characteristics is known as feature extraction; need to select a subset of characteristics that contributes more to the best classification, hence features should be prioritized according to their importance in the classification problem.

#### Feature selection algorithm

The following algorithm was used for feature extraction in this study.

Let *y* ∈ *R*^64^ be a feature vector of the data matrix and *z* ∈ *R*^64^ be the same feature vector in the transformed domain.

$$z_{i}=\alpha \frac{y_{i}-y_{min}}{y_{max}-y_{min}}-\beta$$ (1)

Where *α* and *β* are constant, and *α* > *β*

We establish maps to segregate z according to the classes. The feature vector z is a noralization form of y.

$$h_{i}:z\to z^{i}$$ (2)

Where *z*_1_ ∈ *R*^8^, *z*_2_ ∈ *R*^10^, *z*_3_ ∈ *R*^26^, *z*_4_ ∈ *R*^10^, *z*_5_ ∈ *R*^10^. Vectors *z*_1_, *z*_2_, *z*_3_, *z*_4_, *and z*_5_ are split of z with respect to the classes labels. Let $\bar{z_{i}}$ be mean of a vector ***z***_*i*_, we define

$$z_{i}{}^{*}=z_{i}-\bar{z_{i}}$$

$$Z_{i}=\frac{1}{N_{I}}\sum_{j=1}^{N_{i}}\left(z_{i}\right)j$$ (3)

For *i* = 1, 2, 3, 4, 5

Where *N*_1_, *N*_2_, *N*_3_, *N*_4_ *and N*_5_ are 8, 10, 26, 10 and 10 respectively.

Now, we concatenate through a non-linear map “g” such that:

$$g:z_{1}{}^{*}\times z_{2}{}^{*}\times z_{3}{}^{*}\times z_{4}{}^{*}\times z_{5}{}^{*}\to X$$ (4)

$$X=\left[z_{1}^{{*}^{T}}|\;z_{2}^{{*}^{T}}|\;z_{3}^{{*}^{T}}|\;z_{4}^{{*}^{T}}|\;z_{5}^{{*}^{T}}\right]$$ (5)

Let *d* be a measure used for the selection of features.

$$d_{i}=\vee_{i}\left(0,\wedge_{j}x_{j}\right)$$ (6)

for *i* = 1, 2,…, 22283

Where *x*_*j*_ is the value in the zero vector. We are doing this for every feature.

#### Training on test data

Consider classes 1,2,3,4 and 5 that have the number of samples n_1_, n_2_, n_3_, n_4_, and n_5_ respectively.


$$C=\left\{1,2,3,4,5\right\}$$

Let ***D***_***i***_ where ***i*** ∈ ***C*** be the corresponding datasets. After feature extraction:

Let F be the set of features extracted. *F* = {f1, f2, f3,…. fk}

We define a mapping h such that:

$$h:\;D_{i}\times F\to \overset{ˇ}{D_{i}}$$ (7)

Where **i** ∈ ***C*** and $\overset{ˇ}{D_{i}}\in R^{k\times n_{i}}$

Consider two datasets $\overset{ˇ}{D_{i}}$ and $\overset{ˇ}{D_{j}}$ such that ***i*** ≠ ***j*** and **i**, **j** ∈ ***C***.

We have to perform two tasks. Firstly, we have to find whether $\overset{ˇ}{D_{i}}$ and $\overset{ˇ}{D_{j}}$ are linearly separable. Secondly, if both are separable then find the separating hyperplanes P_ij_. For Linear separability: We define

$$\overset{ˇ}{D_{i}}=\left[{\tilde{d}}_{1}^{i}\;{\tilde{d}}_{2}^{i}\ldots ‥{\tilde{d}}_{n_{i}}^{i}\right]$$ (8)

$$\overset{ˇ}{D_{j}}=\left[{\tilde{d}}_{1}^{j}\;{\tilde{d}}_{2}^{j}\ldots ‥{\tilde{d}}_{n_{j}}^{j}\right]$$ (9)

Both classes i and j are linearly separable if ***f***_***x***_ ∈ *R*^*k*^ and b ∈ R1 such that:

$$X^{T}{\tilde{d}}_{u}^{i}-b\begin{array}{c} \tilde{D_{i}}\\ ≷\\ \tilde{D_{j}} \end{array}X^{T}{\tilde{d}}_{v}^{j}-b$$ (10)

∀ *u* ∈ {1,2,.…. *n*_*i*_} and ∀ *v* ∈ {1,2,.…. *n*_*j*_}

We model this problem as an optimization. The Problem is given as follows:

$$X^{T}{\tilde{d}}_{u}^{i}-b\geq 1$$ (11)

$$X^{T}{\tilde{d}}_{v}^{j}-b\leq -1$$ (12)

for ∀ *u* ∈ {1,2,.….*n*_*i*_} and ∀ *v* ∈ {1,2,.….*n*_*j*_} (11) and (12) is

$$0\geq -X^{T}{\tilde{d}}_{u}^{i}+b+1$$ (13)

and

$$0\geq X^{T}{\tilde{d}}_{v}^{j}-b+1$$ (14)

We introduce variables as *y*_*i*_ ≥ 0 (*i* = 1,2,.…. *n*_*i*_) and *y*_*j*_ ≥ 0 (*j* = 1,2,.…. *n*_*j*_)

Such that

$$y_{i}\geq -X^{T}{\tilde{d}}_{u}^{i}+b+1$$ (15)

and

$$z_{j}\geq X^{T}{\tilde{d}}_{v}^{j}\;-b+1$$ (16)

Writing these equations in the matrix form:

$$Y\geq -X^{T}\overset{ˇ}{D_{i}}+1_{n_{i}}b+1$$ (17)

$$Z\geq X^{T}\overset{ˇ}{D_{j}}+1_{n_{j}}b+1$$ (18)

Vector **1**_**a**_ has **1** a-times.


$$Y={\left[y_{1},y_{2},.\ldots .y_{n_{i}}\right]}^{T}$$ (19)

$$Z={\left[z_{1},z_{2},.\ldots .z_{n_{j}}\right]}^{T}$$ (20)

We introduce constraints in the standard form of an LP from the above equations

$$\left[\begin{array}{cc} \overset{ˇ}{D_{i}} & 1_{n_{i}}\\ \overset{ˇ}{D_{j}} & 1_{n_{j}} \end{array}\right]\left[\begin{array}{c} X\\ b \end{array}\right]\leq \left[\begin{array}{c} Y\\ Z \end{array}\right]$$ (21)

$$-y_{i}\leq 0\;\left(i=1,2,\ldots ,n_{i}\right)$$

$$-z_{i}\leq 0\;\left(j=1,2,\ldots ,n_{i}\right)$$

The objective function is defined as:

$${min}_{y_{i}z_{j}\;X\;b}\frac{1}{n_{i}}\begin{array}{c} n_{i}\\ \sum \\ i=1 \end{array}y_{i}+\frac{1}{n_{j}}\begin{array}{c} n_{j}\\ \sum \\ j=1 \end{array}z_{j}$$ (22)

#### Planes testing

Let ***P***_*ij*_ be a place between class **i** and **j** (***i*** ≠ ***j and j*** ∈ ***C***). The list of planes is given below in Table 3.

**Table 3:** Planes discriminating between the class i and j.

	**1**	**2**	**3**	**4**	**5**
**1**		P_12_	P_13_	P_14_	P_15_
**2**			P_23_	P_24_	P_25_
**3**				P_34_	P_35_
**4**					P_45_

Let $\tilde{D}=\;\left[{\tilde{D}}_{1}\;{\tilde{D}}_{2}\;{\tilde{D}}_{3}\;{\tilde{D}}_{4}\;{\tilde{D}}_{5}\right]$

$$y_{ij}=\tilde{D}\;P_{ij}^{T}$$

Let $Q_{ij}^{*}$ be an optimal threshold that gives maximum correct binary classification decisions for plane ***P***_*ij*_ for the entire data from ***y***_*ij*_. These are considered as bias for the plane ***P***_*ij*.s_

$${\tilde{P}}_{ij}={\left[P_{ij}^{T}\;Q_{ij}\right]}^{T}$$ (23)

## Results

In this section, we provide our findings on leukemia subtypes classification using microarray data. Our method is based on linear programming. The best selected of the observed features are those that are extremely clearly distinct, and these features are more useful for classification and diagnosing the various subtypes of leukemia. From the dataset of 22283 features, we have chosen those features that satisfied the following two goals: 1. Distinguish those traits that make it easier to data separability. 2. Decide which qualities are most useful for classifying new data. Leukemia 64 samples overall, comprising 8 instances of Bone Marrow CD34, 10 cases of Bone Marrow, 26 cases of AML, 10 cases of PB, and 10 cases of PBSC CD34, are included in our dataset. The feature size of our dataset, 22283 genes’ expression levels, is included in the dataset. The gene expression profiles’ values are contained in these characteristics (GEP). Each of these characteristics aids in placing a sample into a certain class. Our dataset includes five distinct classifications or subtypes of leukemia. The proposed model utilizes these five categories—Bone Marrow CD34, Bone Marrow, AML, PB, and PBSC CD34—to identify the class to which each sample belongs. The 22283 features in our dataset contribute significantly to the curse of dimensionality, which is caused by small sample sizes relative to huge numbers of characteristics. When we compute such data, the curse of dimensionality will require our time, memory, and effort. The selected extracted features details have been given in Table 4.

**Table 4:** Details of extracted features after feature selection.

Sr. No.	Feature No.	Probe Set ID	Sr. No.	Feature No.	Probe Set ID
1	5436	205909_at	14	12159	212774_at
2	1756	202228_s_at	15	2899	203372_s_at
3	9888	210410_s_at	16	1951	202423_at
4	2784	203257_s_at	17	18418	219054_at
5	8673	209179_s_at	18	7538	208029_s_at
6	6863	207339_s_at	19	4881	205354_at
7	5699	206173_x_at	20	17796	218431_at
8	5294	205767_at	21	15603	216231_s_at
9	3831	204304_s_at	22	4048	204521_at
10	10839	211429_s_at	23	2539	203010_at
11	7616	208107_s_at	24	1712	202184_s_at
12	9537	210052_s_at	25	17618	218253_s_at
13	188	200660_at			

Feature Number is the serial number of features in the whole dataset as the dataset contains 22283 features in total. Probe Set ID is the label of each feature in the used dataset. It is also a unique number allotted to a specific and relevant group of genes in genetic engineering. The results of 25 selected features are drafted in a table given below in Table 5.

**Table 5:** Distribution of testing and training samples.

Sr. No.	Class	Total Samples	No. Training Samples	No. Testing Samples
1	bm34	8	5	3
2	bm	10	6	4
3	aml	26	16	10
4	pb	10	6	4
5	pbsc34	10	6	4
	Total	64	39	25
	**Percentage**		60.94	39.06

The total number of samples in each class and divided into testing and training samples. The training and testing samples provide 60% and 40%, respectively. Pairwise precision classification on training and testing samples yields 100% and 98.44% accuracy, respectively. Pairwise precision for testing samples have been discussed in Table 6.

**Table 6:** Pairwise precision for testing samples.

	**Class 1**	**Class 2**	**Class 3**	**Class 4**	**Class 5**
**Class 1**		4/7	12/13	7/7	7/7
**Class 2**			11/14	5/8	6/8
**Class 3**				13/14	12/14
**Class 4**					8/8

Table 7 describes, pairwise classification. Class 1 is initially evaluated against classes 2, 3, 4, and 5. Then class 2 is tested using (class 3, class 4, and class5). Then Class 3 is examined using (Class 4 and Class 5). Finally, Class 4 and Class 5 are put to the test. Precision is calculated at each stage of this classification. Class 1 & 2 are initially assessed with pairwise classification by combining two classes (class 3, class 4, and class 5). Then class 3 is tested using (class 4 and class5). Then Class 4 is compared to Class 5. Table 7 depicts the gene expression levels of 22283 genes from 64 samples (rows).

**Table 7:** Pairwise precision merging classes 1 & 2.

	**Class 1&2**	**Class 3**	**Class 4**	**Class 5**
**Class 1&2**		17/17	11/11	11/11
**Class 3**			13/14	12/14
**Class 4**				8/8

Table 8 lists the pairwise classification plane values. We analyzed the performance of pairwise classification, which was initially developed to reduce multi-class issues to two-class problems. Paired classification is also advantageous for computationally expensive learning approaches. Instead of initially attempting to arrange the items, pairwise comparisons between the individual items and later adding the wins for each item make it simpler for a human to discern the order between the n items.

**Table 8:** Pairwise classification planes.

	1 and 2	1 and 3	1 and 4	1 and 5	2 and 3	2 and 4	2 and 5	3 and 4	3 and 5	4 and 5
1	3.1244	61.8378	39.2539	11.5041	51.0343	6.42300	10.7297	8.8339	-50.0586	-2.5127
2	-11.4367	-26.2642	13.0689	-3.3174	-5.2706	-3.0783	-11.3172	22.2851	-8.3967	4.7620
3	-3.5294	-28.8702	-3.1665	-2.3161	-21.6040	1.8454	8.0890	-5.0484	-14.8895	-4.4781
4	-10.4274	-6.02137	1.6154	-7.8471	-2.1069	1.3734	-7.7901	12.8375	-31.7937	-0.5638
5	0.6142	-1.4430	-30.6832	2.0634	7.9035	-3.5018	0.3548	-1.8893	-5.7689	-2.1615
6	6.3294	21.0053	-4.3968	-11.5077	-5.4416	-3.2599	-21.3269	-25.0266	-20.5654	6.9908
7	38.2879	7.9505	-5.4094	17.0848	-18.6942	1.9290	7.3806	-10.3947	-39.8698	5.0184
8	-11.4736	14.2878	3.9398	3.6352	21.1232	7.3946	-0.3406	-4.6731	-10.6660	0.1935
9	31.4478	9.6118	8.8253	-10.3817	28.1867	2.6376	-15.2232	6.35530	-18.2779	-11.4687
10	-10.2388	-10.4577	5.7476	8.5943	14.8300	-8.1539	4.4779	9.5029	-5.6327	2.6915
11	12.9286	-14.5717	-8.2801	-3.8673	-44.4263	-2.6262	-10.7585	-10.7799	29.1867	-0.9146
12	11.9772	-5.82857	21.8163	23.9959	15.7306	2.3577	30.9130	10.1160	29.2573	2.6274
13	15.1716	0.9966	-10.2325	0.0807	-26.4200	-3.4071	-15.726	-12.7050	-2.7461	6.3260
14	50.7243	28.0043	10.3242	5.9381	9.4689	-3.8966	3.3775	-17.6009	-9.0542	-0.5204
15	-34.3428	-13.3798	2.8392	-10.7951	8.0422	0.6620	2.2041	15.7283	-10.7753	-0.6047
16	11.4853	21.5102	-10.2572	-11.5979	-21.9283	-5.1328	-11.4570	-38.1391	-6.25925	2.62015
17	-71.6884	-20.2051	3.2891	-24.9248	-9.4188	-0.9843	-21.9925	13.5433	-13.4275	-2.1903
18	0.2187	2.9279	-6.4697	-4.0762	6.0557	2.4159	6.7933	1.8917	4.1904	-10.3378
19	3.8289	14.8631	10.5697	15.7135	7.2942	4.6883	17.9827	-10.9486	14.4910	0.0136
20	-2.0963	-5.0891	-9.7107	3.8446	21.0788	1.5058	0.9746	5.3162	26.7089	3.6531
21	-24.2060	-23.4062	-8.9004	2.85252	-28.3333	1.1926	9.7745	10.4239	37.2917	3.5650
22	-4.3082	-22.9740	-8.8559	3.5569	-12.2964	3.7189	7.1926	-2.4879	-35.0919	-3.9536
23	-26.7921	-21.4716	-11.3182	-11.1686	-14.8512	-3.1198	-6.2324	3.4180	72.2127	-1.2264
24	43.8786	36.1159	8.5415	9.2864	26.2906	3.4352	6.1964	2.0361	-28.1897	-0.3494
25	7.5024	-4.7042	7.1384	5.7778	7.0883	3.0035	9.2259	19.5262	49.6161	-3.1823

Table 9 shows the pairwise classification plane values obtained by combining Class 1 and Class 2. These plane values are employed in the classification of testing samples following class merging. As previously explained, pairwise classification yields binary classification, which reduces a multi-class problem to a two-class problem.

**Table 9:** Planes with the merge of classes 1 & 2.

	1&2 and 3	1&2 and 4	1&2 and 5	3 and 4	3 and 5
1	94.75262757	38.34882128	11.98566179	8.833862446	-50.05856415
2	-18.01592524	13.31333946	2.419514723	22.28515032	-8.396727361
3	-37.98394979	0.732743278	8.232753611	-5.048367879	-14.88947099
4	7.704706271	2.526028482	-11.45562286	12.83752481	-31.79373956
5	-5.470468391	-31.15365072	4.241484067	-1.889274205	-5.768871167
6	4.172524122	-4.663958474	-21.57306557	-25.02657048	-20.56541405
7	-31.7683911	-10.90283359	6.570915365	-10.39477755	-39.86973388
8	14.15124674	2.651576227	3.151332224	-4.673119429	-10.66605601
9	15.56407367	8.157596034	-30.10568775	6.355306764	-18.27798895
10	12.11494138	4.492564205	10.11693758	9.502911795	-5.632680853
11	-28.63691352	-8.746253864	-6.196700896	-10.77995014	29.18674664
12	15.9528112	20.01355082	29.08587534	10.1159685	29.25733762
13	-23.87257306	-7.817423329	-7.130850936	-12.70504076	-2.746050658
14	35.29731149	10.58104544	8.297171697	-17.60093318	-9.054231139
15	5.241620033	6.744763478	3.650021542	15.72832743	-10.77531427
16	-22.4923462	-11.88216152	-15.41174713	-38.13907173	-6.259253639
17	1.198640153	6.564476244	-19.24315647	13.54331814	-13.4275936
18	5.358345377	-3.724553993	2.157235654	1.891679154	4.190426522
19	7.891845459	10.47554572	13.07832065	-10.94858906	14.49096972
20	-2.988935173	-11.32228711	-2.481191346	5.316202118	26.70894327
21	-28.77003126	-8.40893431	11.08288805	10.42392162	37.29173511
22	-22.09872293	-9.105680321	12.40431993	-2.4879309	-35.09196265
23	-18.06424549	-11.18442352	-14.27264422	3.418014227	72.21271097
24	35.51943456	5.812231525	2.357220844	2.036074664	-28.18970599
25	4.496192489	8.419067409	8.129776209	19.52625135	49.61607088

Table 10 shows binary classification with planes and a threshold. It includes Leukemia used as a threshold for classes represented by numbers 1,2,3,4, and 5. Ҩ is used as a threshold. P_No_ stands for Plane Number. Output classification demonstrates class partition.

**Table 10:** Binary classification using planes with thresholds.

Classes	Ҩ	P_No_	Output classification
{1,2,3,4,5}	0	6	{1,3,5}{2,3,4}
{1,3,5}	0	3	{1,3}{3,5}
{2,3,4}	-50	4	{2,3}{3,4}
{1,3}	70	2	{1}{3}
	-85	6	{3}{5}
{2,3}	0	1	{2}{3}
{3,4}	75	6	{3}{5}

**Fig 2:**
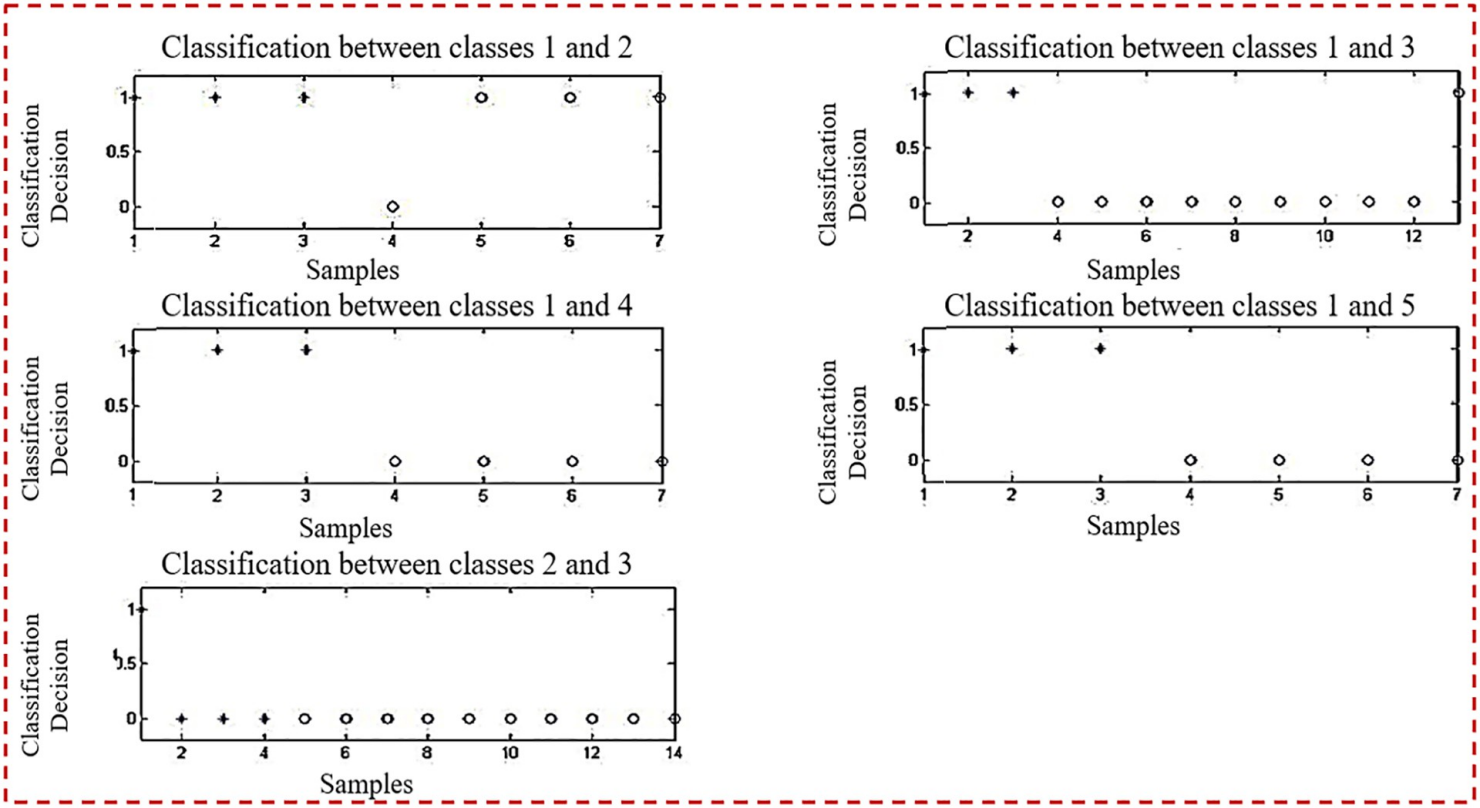
Classification of testing samples using 10 planes (given in Table 8).

Fig 2 depicts the categorization of testing samples utilizing ten planes at the same time. It discusses the pairwise categorization of classes by matching them. Identify misclassified samples as well. Fig 2, the first half of the picture, shows five sub-images.

**Fig 3:**
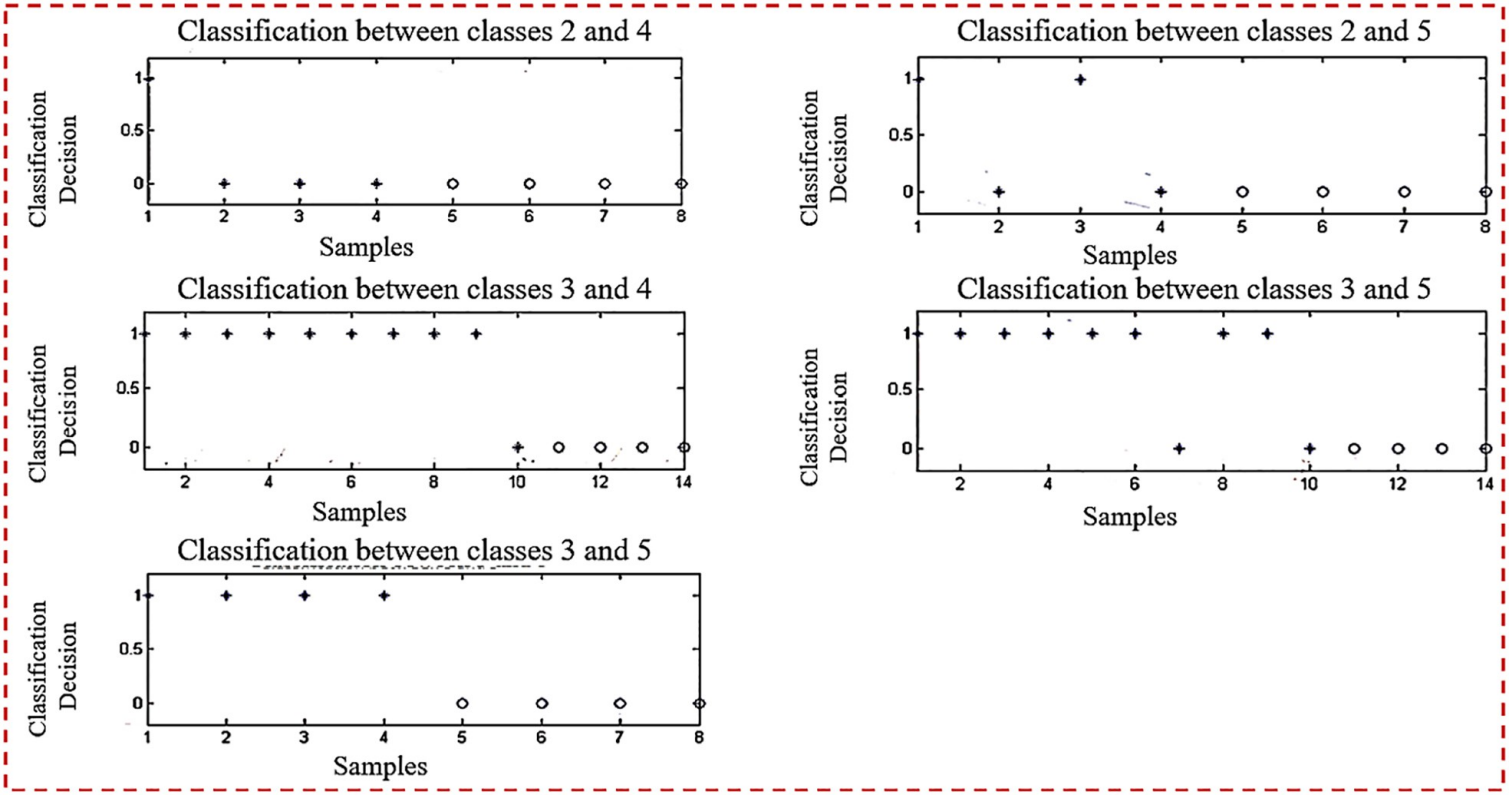
Classification of testing samples using 10 planes.

Fig 3 depicts the categorization of testing samples utilizing ten planes at the same time. It discusses the pairwise categorization of classes by matching them. Identify misclassified samples as well. Fig 3, the second half of the whole picture, shows five sub-images.

**Fig 4:**
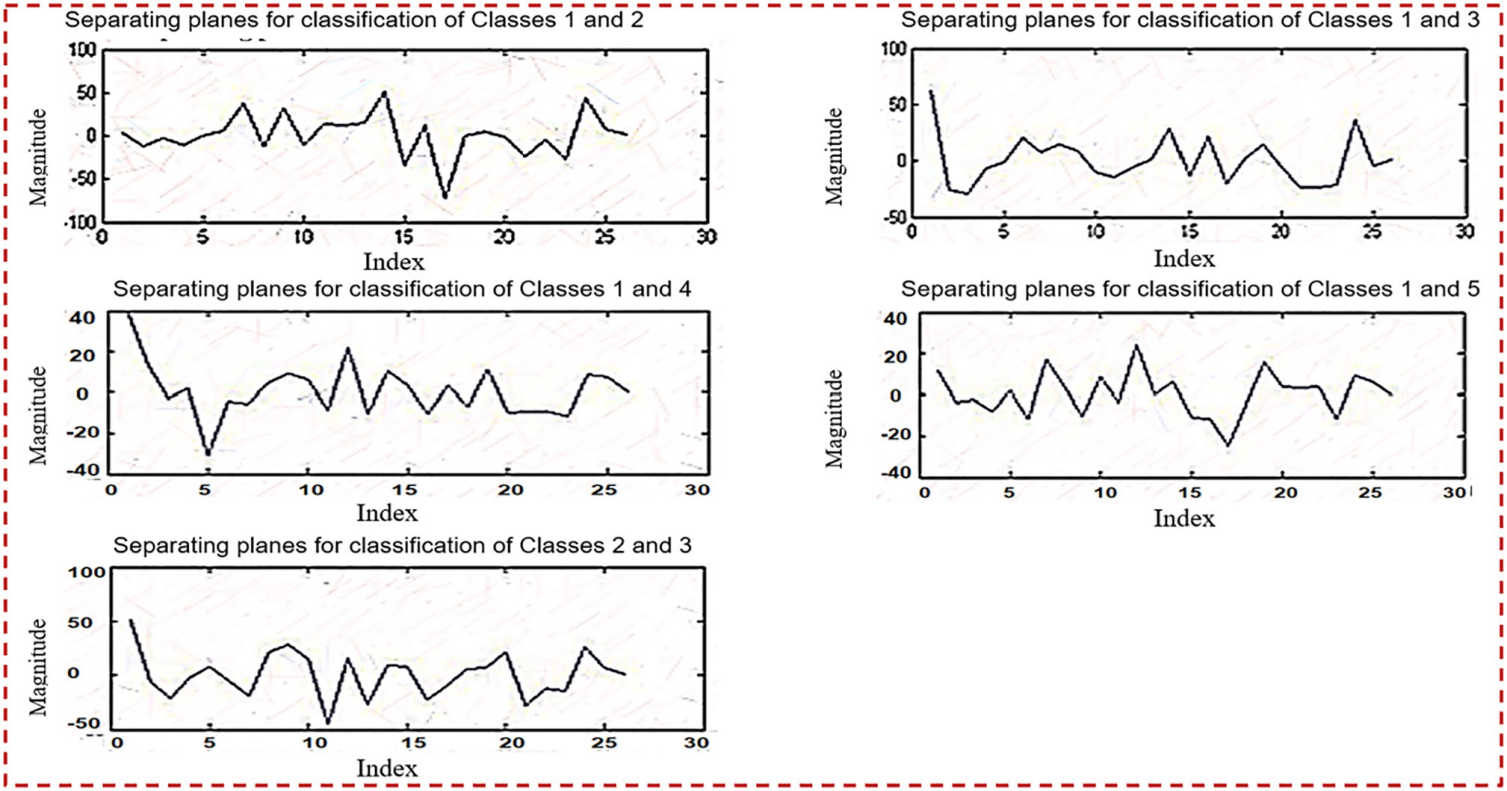
Planes used for binary classification.

Fig 4 depicts planes that are utilized for binary classification. The objective of binary classification is to divide the items of a set into two groups (each termed class) based on a classification rule. Fig 4 depicts five sub planes in the first half of the Figure.

**Fig 5:**
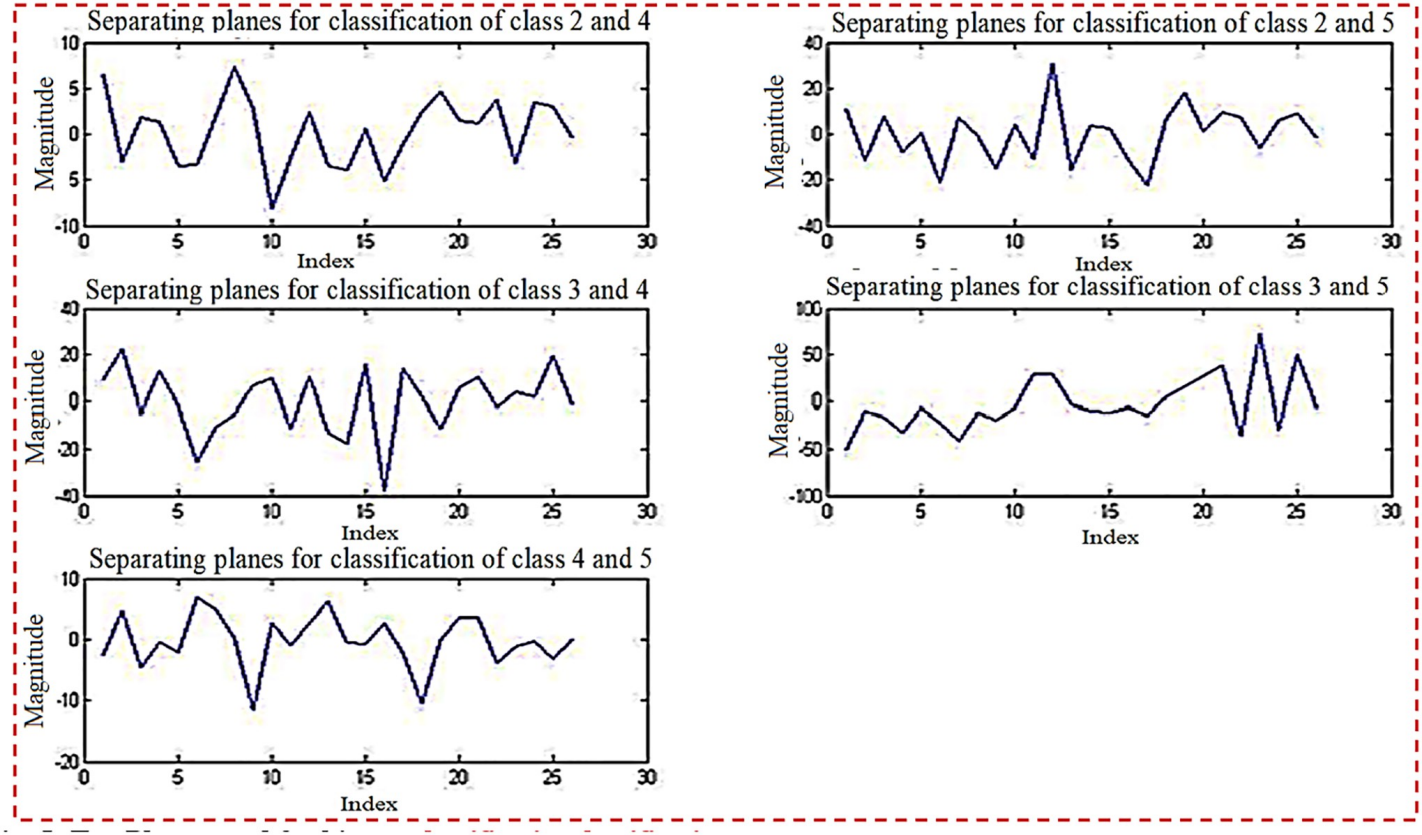
Ten planes used for binary classification.

Fig 5 depicts planes that are utilized for binary classification. Fig 5 depicts five sub planes in the first half of the picture.

**Fig 6:**
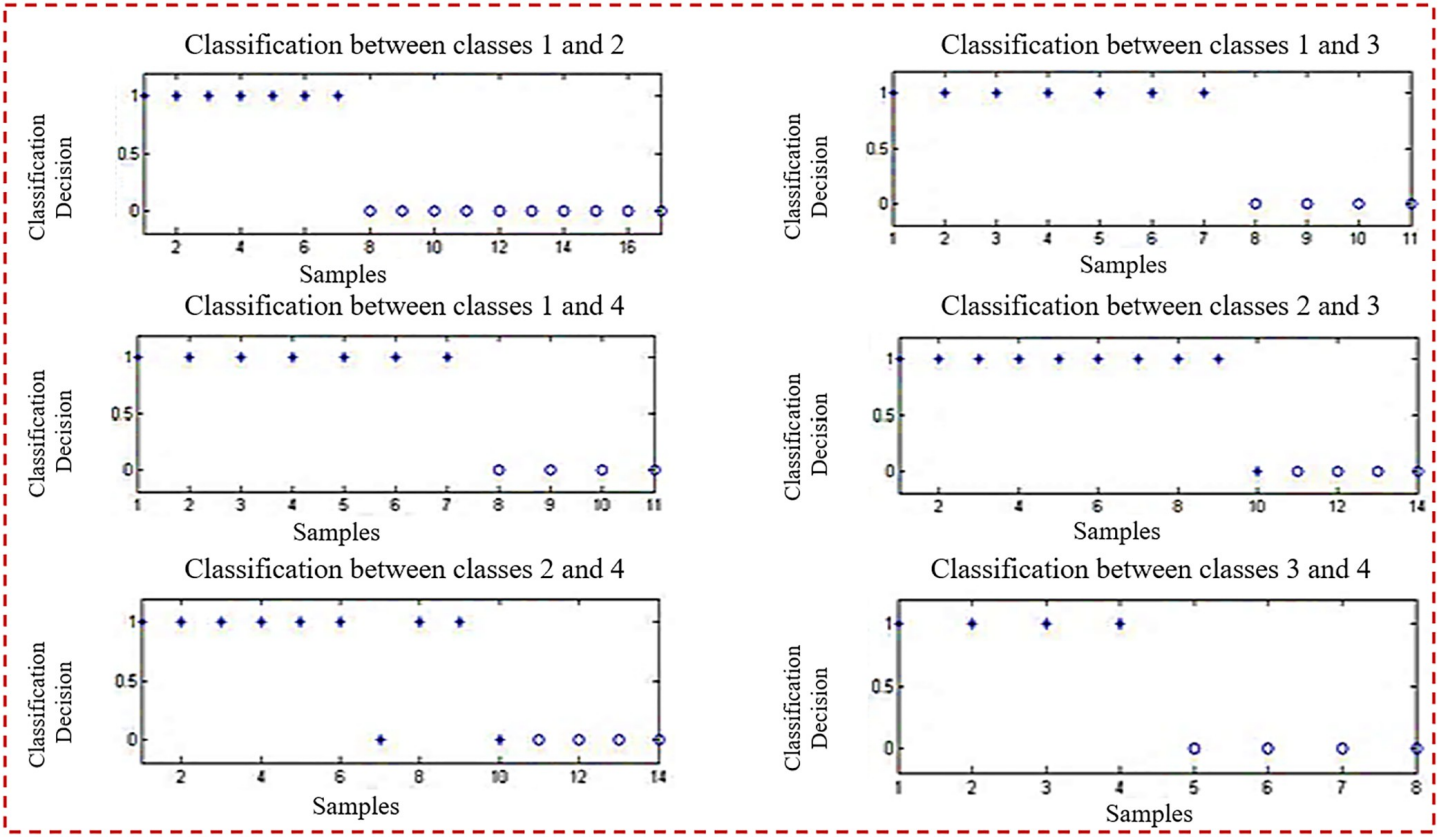
Classification of testing samples using 6 planes.

Fig 6 depicts the categorization of testing samples utilizing six planes at the same time. It discusses the pairwise categorization of classes by matching them. Identify misclassified samples as well. The table contains a list of related samples (Table 9).

**Fig 7:**
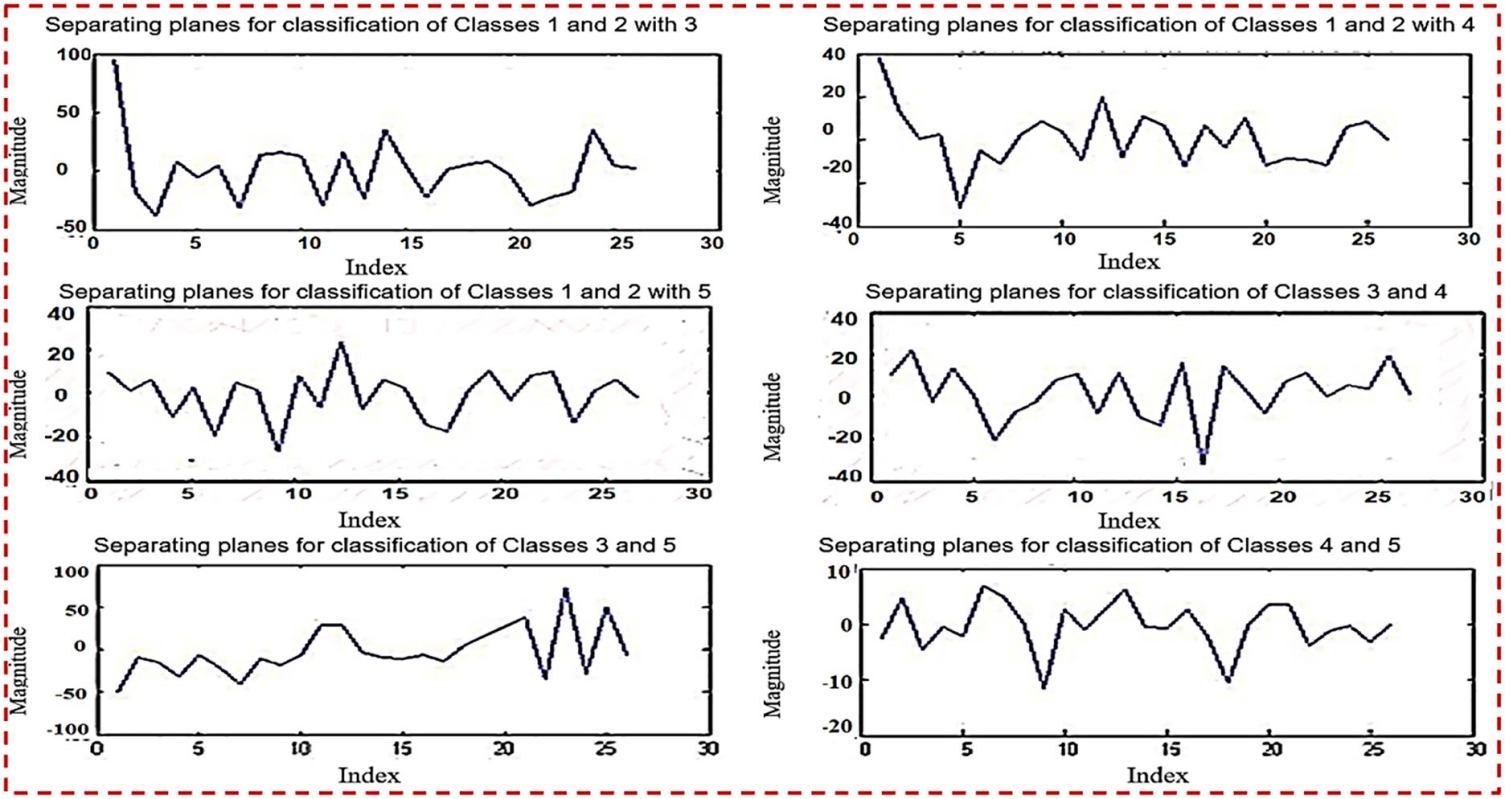
Six planes used for binary classification.

Fig 7 depicts planes that are utilized for binary classification. This is done by merging two classes and comparing them with all other classes. The objective of binary classification is to divide the items of a set into two groups (each termed class) based on a classification rule.

**Fig 8:**
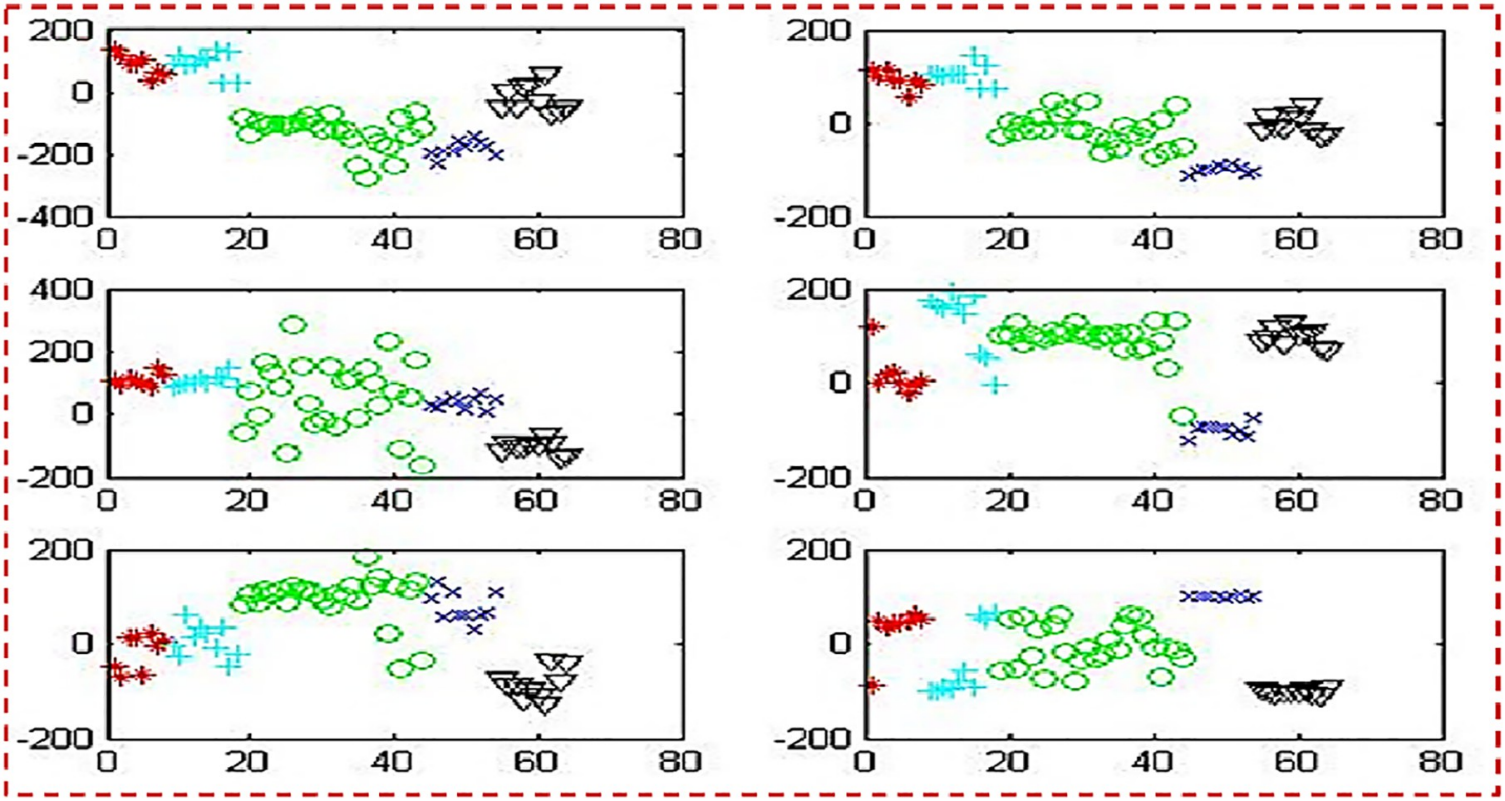
Projections of testing samples on 6 planes.

Fig 8 employs five colours: red, green, blue, cyan, and black. These colours were used in the projection of testing samples. On planes, this colour scheme successfully distinguishes 5 classes and emphasizes their role in classification.

**Fig 9:**
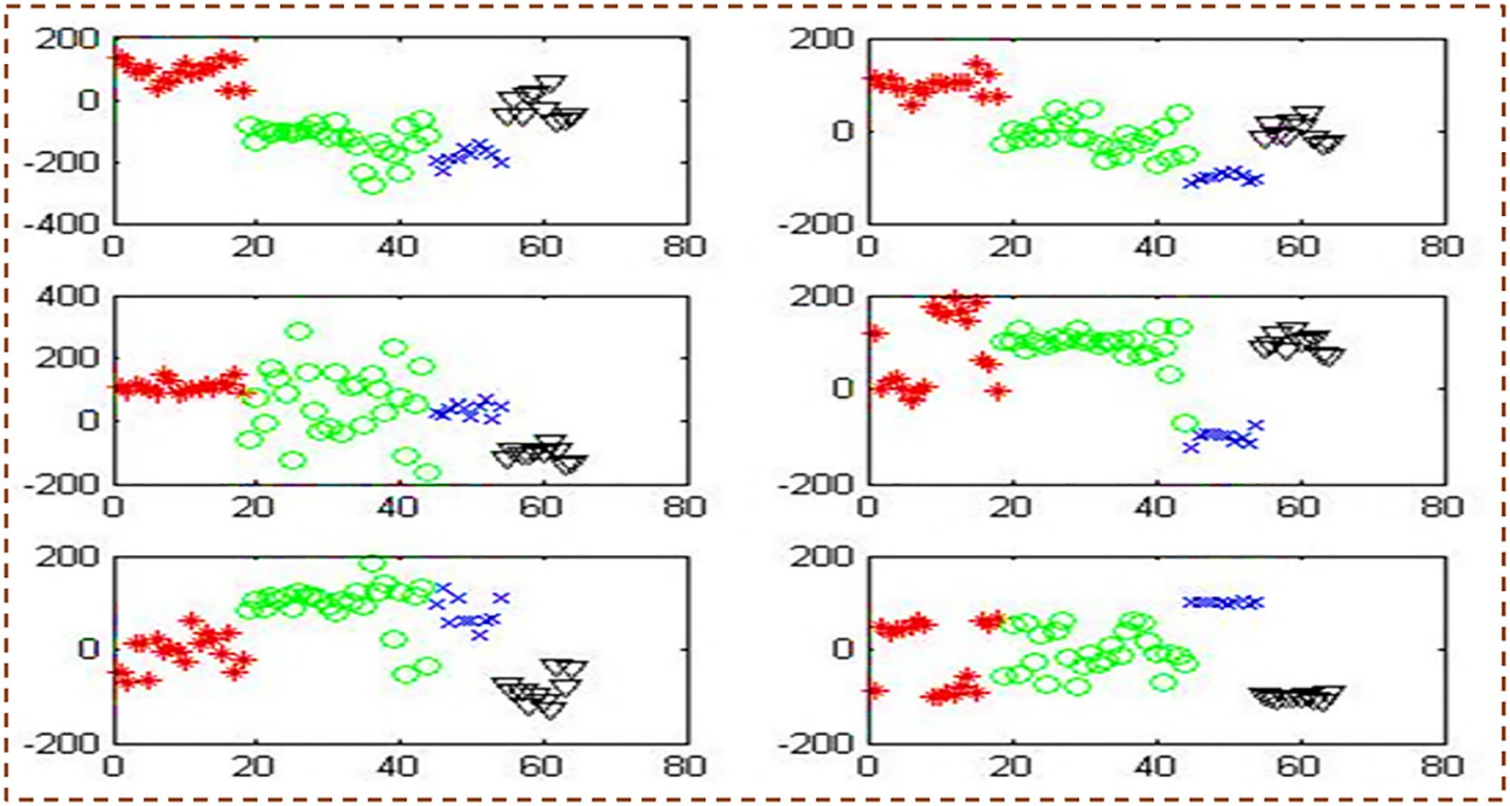
Projections of testing samples on 6 planes merging classes 1& 2.

Fig 9 employs four colours: red, green, blue, and black. Here red colour used for two merging classes (classes 1& 2). Total of 4 classes were used in the projection of testing samples. On planes, this colour scheme successfully distinguishes 4 classes and emphasizes their role in the classification.

Pairwise precision classification on training samples yields 100% accuracy. Pairwise precision classification on testing samples gives 98.44% accuracy. We improved our results by extracting data separation features. Reduced feature count improved prediction performance in terms of accuracy as well as speed. The confusion matrix is given in Table 11 below.

**Table 11:** The confusion matrix value.

Predicted Values					
**Actual Values**		**Class 1& 2**	**Class 3**	**Class 4**	**Class 5**
	**Class 1& 2**	**17**	**1**	**0**	**0**
	**Class 3**	**0**	**26**	**0**	**0**
	**Class 4**	**0**	**0**	**10**	**0**
	**Class 4**	**0**	**0**	**0**	**10**

The accuracy, precision, recall, and F1 score have been given the Table 12.

**Table 12:** The accuracy, precision, recall, and F1 score.

	Precision	Recall	F1 Score
**Class 1& 2**	0.94	1.0	0.97
**Class 3**	1.00	0.96	0.98
**Class 4**	1.00	1.00	1.00
**Class 4**	1.00	1.00	1.00
	Average = 0.985	Average = 0.99	Average = 0.9875
**Accuracy: 98.44**			

The comparison results of the proposed model have been discussed in Table 13.

**Table 13:** Comparisons of results with the existing.

Ref	Dataset	Study Area	Methodology	Performance Measure	Results
Silva et al. (2021)	Gene Expression Omnibus (GEO)	Diagnose AML and ALL leukemia types.	ML Ensemble	94%	Better classification
Abdul Karim (2022)	Gene Expression Omnibus (GEO) GSE9476	Leukemia Cancer Classification	decision tree (DT), naive Bayes (NB), random forest (RF) machine (SVM)	98%	classify logistic regression at optimum speed and accuracy
Grisci et al., (2019)	Gene Expression Omnibus (GEO) GSE9476	Pattern Identification in Cancer Research	FS-NEAT	93% approx.	Improved the performance of algorithms.
**Proposed Model**	Gene Expression Omnibus (GEO) GSE9476	Classification of Subtypes of Leukemia	Linear programming	98%	Improve Accuracy & Better Classification

## Conclusion and future work

Targeting particular treatments for various categories of leukemia patients is one of the biggest medical problems. Improvements to classification models have made them crucial for better cancer treatment. In this work, Linear programming computational models were used to establish the diagnosis of various leukemia subtypes, such as Bone Marrow CD34, Bone Marrow, AML, PB, and PBSC CD34. Leukemia gene expression data from CuMiDa was employed. To make our diagnosis computationally fast, we first rescaled the dataset’s 22283 features and then we chose the most important features technique. The most significant 25 features were selected that have high discrimination power. This study improved the accuracy of the dataset by 98%. Linear Programming models play an important role in the classification of leukemia subtypes. Our model’s overall performance was outstanding. This work contributed to the revelation that when leukemia subtypes are accurately classified and data is fitted with high classified accuracy, cure rates increase and unnecessary toxicities decrease. Because the patient will be able to take preventative measures and doctors will be able to spot the condition earlier. In the future, we can predict such data that have more samples and more subtypes of leukemia. So those types that are not addressed in this study can also be addressed. The expansion of datasets, which will give us access to more samples in the future, brings with it some new challenges. We are able to predict more accurate and complicated classifiers. Reducing the number of created classifiers while using a large number of them simultaneously, as is the case with ensembles of classifiers, is thus one of the primary goals for the future. It is challenging to consistently and precisely classify cancerous cells while avoiding overfitting because of a lack of data, digitization problems, and the curse of dimensionality.
